# 

**Published:** 1891-02

**Authors:** 


CONTENTS.

VOL. 38. No. 2.

| | PAGE. |
|---|---|
| Mind Reading | 25 |
| The Hygiene of Motherhood | 26 |
| How to Look Well | 30 |
| A Remarkable Case | 31 |
| Professor Swing on Immortality | 32 |
| Water at Meals | 33 |
| Convulsions | 34 |
| Headache and the Eyes | 34 |
| Too Many Meals | 35 |
| Concerning Boils | 36 |
| Five Ways to Cure a Cold | 36 |
| A New System of Cure | 37 |
| The Term Microbe | 38 |
| What Rang the Telephone Bells? | 39 |
| The Use of Drugs | 40 |
| Baldness | 40 |
| Miscellaneous | 41 |
| Clairvoyance Extraordinary | 42 |
| Deadly Poisoned Arrows | 43 |
| Literary | 46 |
| A Maxim | 48 |

INDEX TO ADVERTISEMENTS OF HALF A PAGE AND OVER.

| | PAGE. |
|---|---|
| Horsford's Acid Phosphate | I |
| Lydia E. Pinkham Med. Co. | II |
| Good Housekeeping for 1891 | III |
| Stonington Line | III |
| Ruckel & Hendel | V |
| Ayer's Sarsaparilla | V |
| Rochester Lamp Co. | VII |
| Emerson Drug Co | VII |
| Herba Vita Remedy Co. | VIII |
| Tribune Chemical Co. | IX |
| Hall's Journal Premiums | X |
| Royal Baking Powder | XI |
| S. Johnson & Co. | XI |

